# Analysis of the Potential Range of *Anticlea sibirica* L. (Kunth) and Its Changes under Moderate Climate Change in the 21st Century

**DOI:** 10.3390/plants11233270

**Published:** 2022-11-28

**Authors:** Nikolai Fedorov, Aliya Kutueva, Albert Muldashev, Alla Verkhozina, Nikolay Lashchinskiy, Vasiliy Martynenko

**Affiliations:** 1 Ufa Institute of Biology, Subdivision of the Ufa Federal Research Centre RAS, Ufa 450054, Russia; 2 Siberian Institute of Plant Physiology and Biochemistry SB RAS, Irkutsk 664033, Russia; 3 Central Siberian Botanical Garden SB RAS, Novosibirsk 630090, Russia

**Keywords:** *Anticlea sibirica*, relict species, MaxEnt, moderate climate change, habitat suitability

## Abstract

The study shows the analysis of the current potential range and the modeling of its changes in the hemiboreal species *Anticlea sibirica*. The models show the habitat suitability for *A. sibirica* under moderate climatic changes (RCP4.5) in the middle and second half of the 21st century. For modeling, we used MaxEnt software with the predictors being climate variables from CHELSA Bioclim and a digital elevation model. The modeling has shown that climate change can be favorable for the spread of *A. sibirica* to the northeastern part of its range by expanding highly suitable habitats in mountainous landscapes along the coast of the Sea of Okhotsk. In the rest of the range, the total area of suitable habitats will decrease. In areas with extremely deteriorating growing conditions, the species will persist in low-competition habitats such as rocky outcrops, riverbanks, and screes. The predicted change in the distribution of *A. sibirica* indicates a possible strong transformation of the vegetation cover in Siberia and the Urals, even under moderate climate change.

## 1. Introduction

*Anticlea sibirica* (L.) Kunth (=*Zigadenus sibiricus* (L.) A. Gray, Melanthiaceae) is a herbaceous perennial bulbous plant. The species grows in Western and Eastern Siberia, the Far East, the Middle and Southern Urals, and northern Mongolia, as well as in China [1], Japan [2], and the Korean Peninsula [3]. Within its main range, as one of the species of the hemiboreal complex, it typically occurs in dry light coniferous forests with deciduous trees of the genus *Larix* Mill. [4]. *A. sibirica* is a relict species of the Pleistocene complex in the Urals and Mongolia [5,6]. In the western part of the range, isolated by the West Siberian Plain, *A. sibirica* is listed in the Red Data Books of the Republic of Bashkortostan [7], the Sverdlovsk [8] and Chelyabinsk [9] regions, and the Perm Krai [10]. The species is also listed on the Mongolian Red List for IUCN [11,12].

The lack of suitable plant communities between the Southern Urals and the Asian part of the West Siberian Plain causes the species to have a discontinuous range. This is common among the ranges of many other Southern Ural Pleistocene relict species of mountain-Asian and Asian origin (such as *Artemisia bargusinensis* Spreng, *Patrinia sibirica* Juss., *Saussurea parviflora* (Poir.) DC., etc.) [5]. These species could have spread from Siberia to the Urals at the end of the Pleistocene through the West Siberian Plain along fluvioglacial and sandy river sediments [5]. However, since pine–birch–larch forests, floristically similar to the hemiboreal forests of Siberia, have been preserved in the Urals, *A. sibirica* could have been more widespread in the hemiboreal pine–birch–larch forests in earlier Pleistocene. These forests’ floristic depletion and final isolation from Siberian forests may have occurred during the warming in the mid-Holocene [13]. Similarly, their isolation may have occurred during this time in other regions where *A. sibirica* is now rare. Thus, this species could mark the distribution of hemiboreal pine–birch–larch forests in the Pleistocene, which is significant since they are now extant in Siberia and the Southern Urals.

Siberia has undergone dramatic climate change in recent decades [14,15]. Since 1850, the mean annual temperature in Siberia has increased by 1–2 °C, while the global average has increased only by 0.5–1 °C; the warming has accelerated since 1990 [16,17,18]. These changes are likely to have strong effects on ecosystems in Siberia, affecting a range of biomes from tundra to temperate steppe which plays a large role in global ecology [19]. It will affect not only ecosystems but also individual species, such as *A. sibirica*, the species growing in relict communities preserved since the Pleistocene.

An isolated part of the *A. sibirica* range is located in southeast Siberia, where warming of 1.4–1.5 °C has been recorded over the last 100 years [20], and there have been no abnormally cold winters since the 1990s. *A. sibirica*, like the other species of the Pleistocene complex, is potentially vulnerable to current and predicted climate changes affecting their isolated habitats [5,21,22]. By now, researchers have already noted the changes in the distribution of coniferous and broad-leaved tree species and plant communities and shifts in the distribution of mountain forests, as well as the decline in the distribution of mountain tundra communities [23,24,25].

The Intergovernmental Panel on Climate Change project (IPCC) [26,27] has developed several scenarios for climate change, one of them being RCP4.5, the moderate climate change scenario. According to the assessment report on climate change in Russia [28], current climate conditions indicate that temperature change is likely to exceed the RCP2.6 scenario. At the same time, the RCP8.5 scenario is considered unlikely [27]. Accordingly, the RCP4.5 stabilization scenario was chosen for this study. Under this scenario, temperatures are projected to increase by 1.4 to 2.7 °C in the middle of the 21st century (2041–2060, hereafter the 2050s) and by 1.8 to 3.3 °C in the second half of the 21st century (2061–2080, hereafter the 2070s) [26]. These changes could cause a decrease in the distribution of relict Pleistocene species, including *A. sibirica*. An abrupt change in the potential range of this species could indicate that climate change exceeds its Holocene fluctuation. The study aimed to analyze the current potential range and to model the changes in the main-range habitat suitability for *A. sibirica* under moderate climatic changes (RCP4.5).

## 2. Results

The resulting model of the current potential range of *A. sibirica* has an AUC of 0.96, which corresponds to a high quality of the model [29]. Table 1 shows the contribution of the variables to the model of the current potential range of *A. sibirica*. Four environmental variables had the highest contribution: temperature seasonality (standard deviation of the monthly mean temperatures) (Bio4), the elevation difference within one pixel (h_max–min_), mean daily air temperatures of the driest quarter (Bio9), and mean monthly precipitation of the warmest quarter (Bio18). The mean diurnal air temperature range (Bio2) had a small contribution to the model but a high permutation importance (38.5%).

### 2.1. Current Potential Range

**Figure 1:**
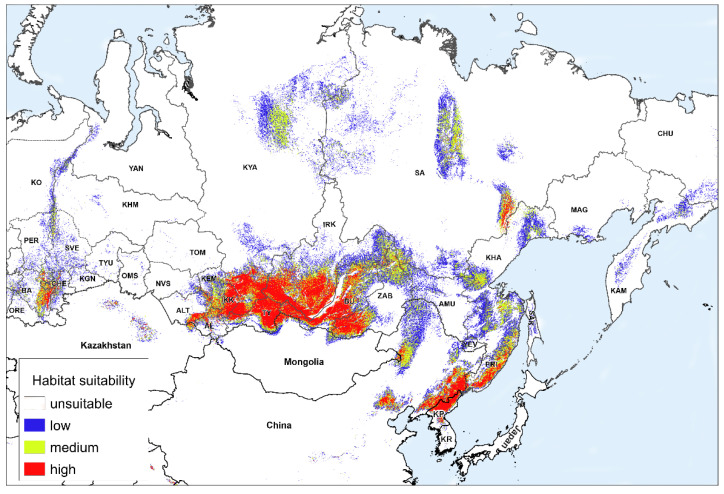
Current potential range of *Anticlea sibirica*. Abbreviated names of countries and Russian regions are given according to the International Organization for Standardization ISO 3166-2 and ISO 3166-2:RU: KP—North Korea; KR—South Korea; AMU—Amur Region; AL—Altai Republic; ALT—Altai Krai; BA—Republic of Bashkortostan; BU—Republic of Buryatia; CHE—Chelyabinsk Region; CHU—Chukotka Autonomous Okrug; IRK—Irkutsk Region; KAM—Kamchatka Krai; KHA—Khabarovsk Krai; KHM—Khanty-Mansi Autonomous Okrug—Yugra; KK—Republic of Khakassia; KYA—Krasnoyarsk Krai; KEM—Kemerovo Region; KGN—Kurgan Region; MAG—Magadan Region; NVS—Novosibirsk Region; OMS—Omsk Region; ORE—Orenburg Region; PER—Perm Krai; PRI—Primorsky Krai; SAK—Sakhalin Region; SVE—Sverdlovsk Region; TOM—Tomsk Region; TYU—Tyumen Region; KO—Republic of Komi; SA—Republic of Sakha (Yakutia); TY—Republic of Tuva Republic; ZAB—Zabaykalsky Krai; YAN—Yamalo-Nenets Autonomous Okrug.

Figure 1 shows the model of the modern potential range of *A. sibirica* with different values of habitat suitability. The habitat suitability is considered only in terms of the bioclimatic and topographic factors included in the study, excluding other possible factors. The threshold value of habitat suitability is 0.41. The potential range map shows habitats of low suitability (0.42–0.60), medium suitability (0.61–0.80), and high suitability (0.81–1.00). Highly suitable habitats in the western isolated part of the range are concentrated in the Southern Urals. In the Middle Urals, they are present in small areas extending along the Ural Mountains up to the Northern Urals. There, the model shows only habitats with low suitability.

According to the model, habitats with low and medium suitability prevail in the northern and middle parts of the Middle Siberian Plateau, which occupies most of the Krasnoyarsk Krai. The northern border of the suitable habitat distribution lies in the north of the Central Siberian Plateau—in the Putorana Plateau and the Anabar Plateau. To the east, in the Republic of Sakha, suitable habitats are mostly confined to the Suntar-Khayata, Verkhoyansk, and Chersky ranges. Highly suitable habitats are more common in eastern Verkhoyansk and on the Suntar-Khayata Range. To the northeast, potentially suitable habitats exist in the mountains on the coast of the Sea of Okhotsk and in Kamchatka. To the south, suitable habitats are common in Khabarovsk and Primorsky Krais, and they also exist in the mountainous part of Sakhalin, in the western part of the Amur Region, and to the south, in China. Highly suitable habitats are widely distributed in the Zabaykalsky Krai, the republics of Buryatia, Tuva, Khakassia, and the Kemerovo Region, as well as in the southern part of the Irkutsk Region, the southeastern part of the Altai Krai, and northern part of the Altai Republic. There, they are confined to the systems of mountain ranges: in the east—to the Sikhote-Alin range, in the west—to the Stanovoy and Yablonovy ranges, and further west—to the Sayan mountains and Altai.

Outside of Russia, suitable habitats are found in the northern mountainous part of Kazakhstan. However, there is no reliable information about the presence of A. sibirica in these habitats. There are small areas with suitable habitats in the mountainous areas in the north of Mongolia, which are the extension of the main habitats of the species in Russia. In addition, suitable habitats are present in the mountainous areas of China, the Korean Peninsula, and Japan.

### 2.2. Climate Change Influence on the Habitat Suitability

**Figure 2:**
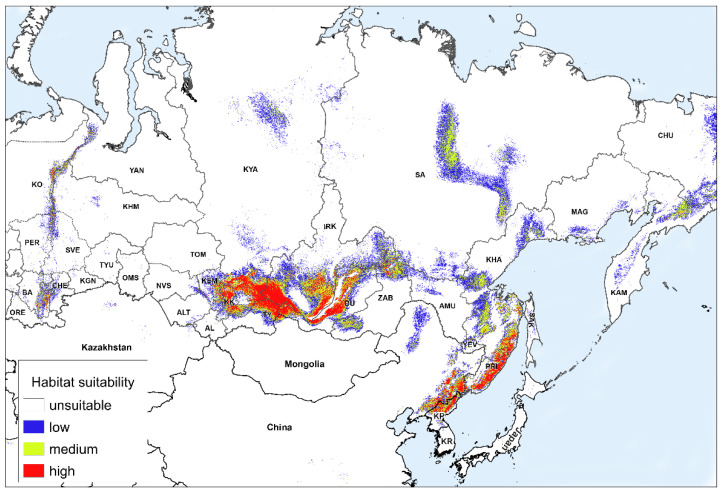
Forecast of habitat suitability of *Anticlea sibirica* under moderate climate change (RCP4.5) in the middle of the 21st century (2050). Abbreviated region names are shown in the notes of Figure 1.

**Figure 3:**
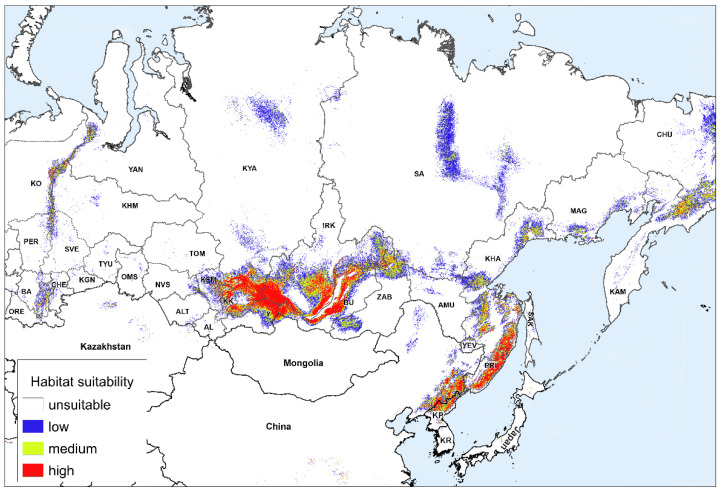
Forecast of habitat suitability of *Anticlea sibirica* under moderate climate change (RCP4.5) in the second half of the 21st century (2070). Abbreviated names of regions are shown in the notes of Figure 1.

The following is a discussion of the impact of climate change on the potential range of the species in Russia. Figure 2 and Figure 3 show the models of habitat suitability of *A. sibirica* in Russia under moderate climate change in the middle (the 2050s) and the second half (the 2070s) of the 21st century. In different parts of the species’ vast range, possible changes in the habitat suitability are expressed in varying degrees, and may sometimes have different directions. Therefore, we will consider separately, the changes in the areas with different habitat suitability in different parts of the range in Russia (Table 2). The regions were divided into three groups according to the type of changes in the areas with different habitat suitability. Group 1 includes the regions with a projected increase in suitable habitats or a slight decrease; group 2 includes the regions with a decrease in suitable habitats in the second half of the 21st century by 20–50%; and group 3 has regions with a decrease of 50% or more. The first group includes the Khabarovsk and Primorsky Krais, as well as the Magadan Region. The Magadan Region is the only region where climate change will result in a simultaneous increase in the areas with low, medium, and highly suitable habitats. The areas with medium and high suitability located near and above the upper boundary of the forest distribution will increase the most. To the south, in the Khabarovsk Krai, the overall distribution of suitable habitats will slightly decrease; however, the areas of highly suitable habitats will increase as in the previous case, especially in the southeastern part of the region. Even further to the south, in the Primorsky Krai, the area of highly suitable habitats will slightly increase, but the distribution of habitats of medium suitability will decrease.

The second group includes seven regions (Table 2). Two of the northernmost and largest regions (Krasnoyarsk Krai and Republic of Sakha) and one mountainous region (Republic of Buryatia) are predicted to have a decrease in the distribution of all groups of habitat suitability. The largest decrease will occur in the northern part of the Republic of Sakha. In all three regions, highly suitable habitats will mostly remain in the most elevated parts of the mountains. For the other four regions located to the south of the Republic of Sakha and Krasnoyarsk Krai (the republics of Khakassia and Tuva, Kemerovo, and Irkutsk regions), the share of areas with habitats of low suitability will increase with the distribution of suitable habitats generally decreasing.

The third group includes eight regions of Russia (Table 2). In four regions of this group, *A. sibirica* is listed in the Red Data Books (Republic of Bashkortostan, Sverdlovsk and Chelyabinsk Regions, and Perm Krai). In the Altai Republic, it is rare but is not listed in the Red Data Book. There are suitable habitats for this species in the neighboring region, the Altai Krai, but it has not been recorded there. With a general decrease in the distribution of *A. sibirica* in another region of this group, Perm Krai, highly suitable habitats increase in the most elevated part, while all suitable habitats decrease in the rest of the region.

All in all, in five regions (republics of Bashkortostan and Altai, Altai Krai, and Zabaykalsky Krai, as well as the Chelyabinsk Region), highly suitable habitats will rapidly decline to the point of near extinction. Thus, the strongest decrease in the distribution of suitable habitats for *A. sibirica* is predicted mainly in areas where this species is currently quite rare due to the lower distribution of suitable habitats.

In Mongolia and Kazakhstan, suitable habitats will almost completely disappear. In China and the Korean Peninsula, they will remain, but habitats of medium and high suitability will occupy smaller areas. In these regions, changes in the areas occupied by suitable habitats were not analyzed, since there was not enough reliable data on the distribution of *A. sibirica* on the altitude gradient, as well as little data about its plant communities.

## 3. Discussion

The analysis of the variables with the highest contribution to the model of the potential range shows the major role of variables characterizing a sharply continental climate: temperature seasonality (Bio4) and mean diurnal air temperature range (Bio2). They both have a high percentage contribution and permutation importance. The difference between the maximum and minimum elevation within a pixel (h_max–min_) characterizes the confinement of the species to mountainous terrain, and this variable also has a high contribution to the model. Within the main range, the species is confined to dry mountainous hemiboreal pine–birch–larch forests, which is reflected in the contribution of the average temperature of the driest quarter (winter) (Bio9) and precipitation of the warmest quarter (Bio18).

At the northeastern border of the species distribution (Magadan Region and Khabarovsk Krai), climate change will increase habitat suitability by shifting the upper border of sparse forest distribution to the subalpine zone. To the south, in Primorsky Krai, a relatively slight decrease in the areas with suitable habitats is predicted, probably due to a decrease in the distribution of light coniferous boreal forests, one of the typical habitats of this species [30]. Thus, along the Pacific coast, climate change will increase the area of highly suitable habitats along a south–north gradient, which coincides with the gradient of increasing climate severity. According to the model, suitable habitats also exist in the mountainous part of Sakhalin Island, where this species has not been recorded [31], yet its single occurrences are theoretically possible, as it exists in mountainous areas of Japan [2]. With climate change, the areas with suitable habitats are predicted to decrease.

In most of Eastern Siberia (the republics of Khakassia, Tuva, Sakha, and Buryatia, as well as Krasnoyarsk Krai and the Irkutsk Region), the areas with suitable habitats are projected to decrease significantly. In addition, this trend will be partly observed in the southeast of Western Siberia (Kemerovo Region). In all of these areas, the model predicts a decrease in the highly suitable habitats as well as in the total area of suitable habitats. In all cases, habitat suitability decreases to a lesser extent in the most elevated parts. In the valleys and lower parts of slopes, climate change will affect hemiboreal pine–birch–larch forests and some other habitats. As a result, the areas of suitable habitats will decrease or completely disappear in some cases, for example, between the Angara River and the East Sayan Ridge (the Irkutsk Region). At the same time, suitable habitats may also remain along steep riverbanks.

The strongest decrease in the area of suitable habitats for *A. sibirica* within the main range is predicted in Zabaykalsky Krai, the Amur Region, and the Republic of Altai, where this species is not widely distributed. There, it is found only in the highlands, on mountain outcrops, and on rock ledges [32,33,34]. Under climate change, habitats of medium suitability will remain only in the most elevated parts of the mountain ranges of these regions, predominantly on rocky riverbanks.

In the western isolated part of the range (the Urals), *A. sibirica* is a rare species. It is found mainly in the Southern Urals (in the Republic of Bashkortostan) and adjacent areas of the Middle Urals (Perm Krai, Sverdlovsk, and Chelyabinsk Regions). In general, the distribution of medium and highly suitable habitats for *A. sibirica* in the Southern Urals matches the distribution of plant communities of hemiboreal pine–birch forests and the range of mixed pine–broad-leaved forests. At the same time, *A. sibirica* does not occur in these forests in the Southern Urals, which may be due to the long period of expansion of broad-leaved tree species in the mid-Holocene [13,35]. Strong coenotic pressure from more closed stands in broad-leaved and mixed pine–broad-leaved forests and their developed undergrowth may have caused *A. sibirica* to disappear from the herbaceous layer. Due to the fact that the modern range of this species is influenced not only by the distribution of the typical plant communities but also by a complex of historical reasons, including climate change, the actual distribution at the range borders is much lower than the areas of potentially suitable habitats. Suitable habitats are also predicted to decrease dramatically in the Southern Urals but remain, and even somewhat increase, in the Middle Urals.

The rate of change in the distribution of *A. sibirica* due to climate change will depend on the rate of seed dispersal and changes in the suitable plant communities. Currently, there are fairly rapid shifts in the upper distribution limits of tree and shrub vegetation [23,36]. In contrast, changes in the ranges of tree species occur at a slower rate than climate change due to their long ontogenetic cycle [37,38]. Accordingly, we can expect that changes in the distribution of *A. sibirica* in the highlands will occur at a faster rate than in the intermountain valleys and lower parts of the slopes.

## 4. Materials and Methods

### 4.1. Studied Species

*Anticlea sibirica* is a herbaceous perennial bulbous plant, 20–80 cm, propagated mainly by seeds [7,10]. It blooms in June–August, and fruits in September. The fruits are 3-celled capsules. The number of plants in the isolated populations is usually low, from a few dozen to a few hundred individuals [7]. At the northern border of distribution in the Southern Urals, the local populations are extremely small, consisting of 10–25 plants, of which only 4–5 plants are generative. This causes the low stability and high vulnerability of these coenopopulations [10]. The studied distribution of *A. sibirica* is the highest in the western part of the range, where the species is included in the Red Data Books [7,8] and significantly lower in the eastern part of the range, especially in the mountain areas with low population density.

The range of this species covers areas with a great diversity of climatic conditions, depending on which species are confined to different types of habitats with different resistance to climatic changes. At the northeastern border of its range, in the Magadan Region, the species is found mainly on the Kolyma Plateau in the subalpine zone of the mountains—in shrub and moss–lichen mountain tundra [39,40]. To the south, in Khabarovsk Krai, *A. sibirica* is found in larch woodlands, stony slopes, cedar shrub thickets, and mountain tundras [41]. In Primorsky Krai, *A. sibirica* is quite rare and it is found on shaded limestone rocks and in mixed-light coniferous forests [30].

In Yakutia, one of the typical habitats of *A. sibirica* is the mountain tundra [42]. It is widely distributed in larch, pine, and mixed herbaceous and humid moss forests, as well as in open forests. They include pine forests confined to gentle slopes of floodplain terraces [43], sparse feathermoss forests with *Larix cajanderi* Mayr in the lower parts of southern mountain slopes and near-valley parts of large rivers [44], as well as in most xerophytic larch shrub–lichen forests with *Larix gmelini* (Rupr.) Kuzen. [45]. In addition, *A. sibirica* may also grow in floodplain meadow communities [46] and cryophilic petrophytic steppes [47].

In Krasnoyarsk Krai, *A. sibirica* is found on the Anabar Plateau, the Putorana Plateau, and in the southern, mountainous part of the region. In the north, the species grows in larch forests, including open larch forests with lichen or moss cover [48]. It also grows in the subalpine belt and mountain tundras. In the southern part of the region, the species grows in pine and larch–pine forests of the taiga type and hemiboreal pine–birch forests [49].

In the Republic of Buryatia, *A. sibirica* grows in the highlands, on turfed slopes of dryad tundras and cryophytic kobresia meadows [50], on rock ledges, in coniferous, larch, and mixed forests, in open larch forests, in glades and yerniks [51]. In the southwestern part of the region, it forms thinned thickets, sometimes over significant areas [51]. In the Republic of Khakassia, it is common in all vegetation belts in the range of hemiboreal pine-birch–larch forests, forest meadows, stone fields, and on rocks [52]. In the Republic of Tuva, *A. sibirica* is quite rare and grows in the forest belt and the lower part of the highlands [53]. In the forest belt, it grows mainly in hemiboreal pine–birch–larch forests [54].

In the Irkutsk Region, *A. sibirica* grows in pine, larch, mixed forests (light coniferous small-leaved and dark coniferous forests), on rocks, slopes, banks of streams, in stone fields, and shrub thickets. In the Kemerovo Region, where the species is listed in the Red Data Book [55], it grows in coniferous, deciduous, and mixed forests, and meadows. It ascends to mountains where it occurs on sodded slopes, rock ledges, and in yerniks. In addition, the species can also grow on stony slopes along riverbanks [55].

In Zabaykalsky Krai, the species is present in light coniferous forests, including forests with *L. gmelini*, and above the tree line. In the Amur Region, *A. sibirica* has a lower distribution than in Zabaykalsky Krai. The species is quite rare and occurs only in the highlands and on residual mountains [32,33]. However, it is not listed in the Red Data Book of this region. In the Altai Republic, this species occurs in the Northern and Central Altai on soddy slopes, rock ledges, forests (including larch open forests), meadows, and yerniks [34]. To the northwest, in the Altai Krai, this species does not occur [56].

In the Urals, *A. sibirica* is a rare species and is found in Perm Krai, Sverdlovsk, and Chelyabinsk Regions, and the Republic of Bashkortostan. In Perm Krai, two localities of this species in feathermoss pine and spruce forests have been recorded [10]. In the Sverdlovsk Region, there are five recorded localities associated with shady limestone rocks of northern and eastern exposition and with rocky screes under the cliffs [8]. In the Chelyabinsk Region, the species grows in pine–birch, broad-leaved dark-coniferous, and larch feathermoss forests, as well as on the cliffs along riverbanks. In the Southern Urals, 79 localities of *A. sibirica* have been recorded in the Republic of Bashkortostan. The species is most widespread in the Ufa Plateau, where it grows mainly on steep northern slopes in the water protection zone of the Pavlovsk Reservoir. There, it grows in feathermoss spruce and larch forests and on steep southern slopes in xerophytic feathermoss pine forests. In the central part of the Southern Urals, the species occurs mainly in shady lower parts of riverside slopes on cliffs and stony screes. The species also very rarely occurs in feathermoss dry pine forests. Thus, the main distribution of the species is associated with plant communities formed under the influence of the continental climate of northern latitudes and highlands.

### 4.2. Species Distribution Modeling

**Figure 4:**
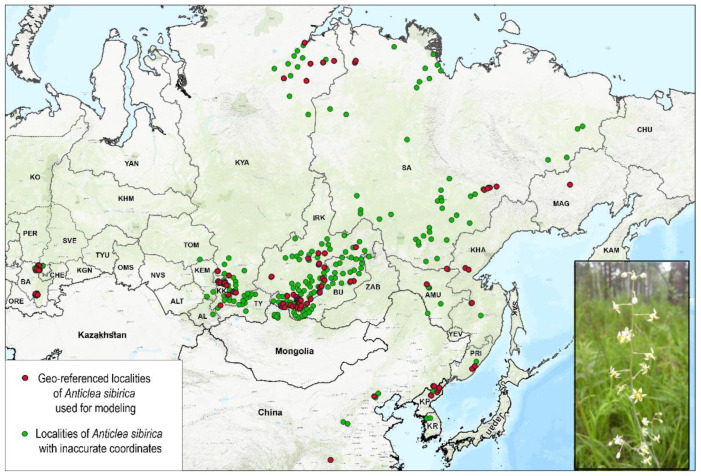
Localities of *Anticlea sibirica* on a topographic map (ESRI Topographic). Abbreviated names of regions are shown in the notes of Figure 1.

The maximum entropy modeling software (MaxEnt v3.4.1k, American Museum of Natural History, Center for Biodiversity and Conservation (New York, NY, USA)) [57] was used to assess the changes in habitat suitability for *A. sibirica* under climate change. The representation of location points from different parts of the species range is important in modeling [58]. To prevent model overfitting due to the high density of georeferenced points in the more studied areas, we used randomly selected habitat points of *A. sibirica* located at a distance of at least 10 km from each other. We used the spThin package in the R software to randomly select localities [59]. After selection, data on 120 georeferenced localities of this species in Russia, China, and the Korean Peninsula were used for modeling. They included localities found in herbarium collections (UFA, IRK), in the Global Biodiversity Information Facility [60], as well as the author’s own data. The accuracy of georeferenced points was up to 100 m, and points with inaccurate coordinates were used in the interpretation of modeling results (Figure 4).

As predictors in the modeling, we used the set of Bioclim climatic variables from the CHELSA database (Supplementary Materials Table S1) [61,62] and the digital elevation model GMTED2010 [63] with a 30 arc sec resolution. The elevation difference was used as a rough estimate of slope steepness. We generated a Pearson correlation matrix of environmental predictors, and in the case of a correlation coefficient greater than or equal to 0.8, one of the variables was excluded to prevent multicollinearity and model overfitting (Supplementary Materials Table S2) [64]. In this case, preference for further use was given to the variables with a larger contribution to the model identified at the preliminary stage. In other cases, we preferred the parameters reflecting quarterly rather than monthly characteristics of temperature and precipitation [65]. Depending on the choice of GCMs, the models may not always produce consistent results. To predict the future distribution of the species, we used an ensemble of four climate change models: CCSM4 [66], NorESM1-M [67], MIROC-ESM [68], INMCM4 [69], which provides a higher accuracy in the results [70]. These GCMs were selected for the ensemble using general guidelines [71,72]. We used the following MaxEnt settings: maximum iterations—400, convergence threshold—0.001, and output format—“cloglog”.

The AUC indicator was used for the statistical evaluation of the model [29]. We applied the “Maximum test sensitivity plus specificity” threshold as the lowest limit for habitat suitability [73]. In the final models, the habitat suitability was divided into three groups: low, medium, and high. The area covered by each suitability level was calculated using QGIS v3.14.1 (QGIS.org, %Y. QGIS Geographic Information System. QGIS Association. http://www.qgis.org (accessed on 2 October 2022)).

## 5. Conclusions

Our research has shown that the climatic niche of *A. sibirica* will shift to higher elevations and toward the north, which is consistent with the results of many studies on other species [74,75]. It is important to note that the greatest range of shifts is expected to happen in the middle of the 21st century. Between 2050 and 2070, the range shifts will be small, and in this period, little loss of potential occurrence areas will be observed compared to the earlier period. This is the first such observation from the area between the Ural Mountains and Siberia, as most studies only focus on 2061–2080. Similar results were recently obtained when studying changes in the potential niche of *Robinia pseudoacacia* L. in Europe [76]. Hence, studies on the impact of climate change on the availability of potential niches should primarily consider the middle of the 21st century, when the magnitude of change is expected to be the highest. With this, our results confirm that the need to develop strategies to mitigate the adverse effects of climate change is more urgent than is commonly believed.

Moreover, future research should also consider the species’ dispersal abilities to estimate the species’ ability to follow the rate of change and phenological shifts. Recent studies [77] show that the rapidly increasing number of observations in citizen science databases and climate maps can help model phenology under current and future climate conditions. Currently, a limitation of such studies for *A. sibirica* is the still small number of records. Nevertheless, future research will allow the niche of the species we are studying and other species to shift in time and space. This will help to gain a complete understanding of ecosystem function and biodiversity in a changing climate in the near future.

## Figures and Tables

**Table 1:** Contribution of environmental variables to the model of the potential range of *Anticlea sibirica*.

Code	Environmental Variable	Percent Contribution	Permutation Importance
Bio4	Temperature seasonality	27.8	23.4
h_max–min_	Difference between maximum and minimum elevation, m	24.4	16.1
Bio9	Mean daily air temperatures of the driest quarter, °C	16.0	7.5
Bio18	Mean monthly precipitation of the wettest quarter, mm	13.6	1.4
Bio2	Mean diurnal air temperature range, °C	11.1	38.5
Bio15	Precipitation seasonality	3.6	3.7
Bio10	Mean daily air temperatures of the warmest quarter, °C	3.5	9.4

**Table 2:** Changes in the areas of different habitat suitability of *Anticlea sibirica* under climate change by 2050s and 2070s in Russian regions as a % of the areas at present.

Regions	Changes in Areas with Different Habitat Suitability under Climate Change, %							
	All Suitability *		Low Suitability		Medium Suitability		High Suitability	
	2050	2070	2050	2070	2050	2070	2050	2070
Regions where the area of suitable habitats is projected to increase or slightly decrease, %								
Magadan Region	95	281	79	200	379	1678	457	13,134
Khabarovsk Krai	−14	−16	−30	−37	−7	−16	211	355
Primorsky Krai	−13	−10	−10	−1	−42	−35	11	7
Regions where suitable habitats are projected to decrease by 20–50 percent, %								
Republic of Khakassia	−21	−26	89	93	43	44	−53	−61
Kemerovo Region	−19	−35	28	10	−53	−64	−43	−66
Irkutsk Region	−22	−19	12	7	−13	−7	−49	−43
Tyva Republic	−50	−44	4	17	−31	−18	−66	−64
Republic of Sakha	−19	−44	−9	−29	−28	−70	−93	−90
Republic of Buryatia	−35	−36	−10	−22	−29	−38	−52	−43
Krasnoyarsk Krai	−30	−30	−27	−30	−23	−26	−39	−32
Regions where suitable habitats are projected to decrease by 50 percent or more, %								
Perm Krai	−57	−65	−62	−71	−44	−50	377	313
Amur Region	−37	−53	−32	−53	−52	−51	−64	−17
Sverdlovsk Region	−68	−72	−69	−73	−71	−73	−37	−46
Republic of Bashkortostan	−44	−54	−13	−19	−53	−66	−77	−89
Zabaykalsky Krai	−59	−59	−34	−32	−50	−54	−92	−91
Chelyabinsk Region	−66	−79	−50	−65	−79	−92	−95	−98
Altai Krai	−88	−88	−70	−70	−96	−94	−100	−100
Altai Republic	−97	−96	−94	−93	−99	−98	−100	−99

* sum of different habitat suitabilities.

## Data Availability

The data that support the findings of this study are available from the corresponding authors upon reasonable request.

## Supplementary Materials

The following supporting information can be downloaded at: https://www.mdpi.com/article/10.3390/plants11233270/s1, Table S1: The estimates of relative contributions of the environmental variables to the MaxEnt model.; Table S2: Correlation analysis of the environmental variables.
