# Supplementary material for: Analysis of the Potential Range of Anticlea sibirica L. (Kunth) and Its Changes under Moderate Climate Change in the 21st Century
Source: Plants (Basel). 2022 Nov 28;11(23):3270. doi: 10.3390/plants11233270 (PMC9738958; doi:10.3390/plants11233270)
Supplement: Supplementary file 1 [file plants-11-03270-s001.zip › plants-1978540-supplementary.pdf]

Table S1. The estimates of relative contributions of the environmental variables to the MaxEnt model.

Code/Unit	Variable	Percent contribution	Permutation importance
**bio4 (°C)**	**Temperature Seasonality**	**22.6**	**9.7**
**$h_{max-min}$ (m)**	**Difference between maximum and mini-mum elevation**	**19**	**10.7**
**bio18 (mm)**	**Precipitation of Warmest Quarter**	**12.5**	**9.5**
**bio2 (°C)**	**Mean Diurnal Range**	**8.6**	**26.7**
**bio9 (°C)**	**Mean Temperature of Driest Quarter**	**8.4**	**4.6**
bio11 (°C)	Mean Temperature of Coldest Quarter	**7.2**	**11.9**
bio3 (%)	Isothermality	6.6	2
**bio15 (CV)**	**Precipitation Seasonality**	**2.7**	**3.4**
bio12 (mm)	Annual Precipitation	**2.7**	1.6
bio19 (mm)	Precipitation of Coldest Quarter	2	0.7
bio7 (°C)	Temperature Annual Range	2	2.5
**bio10 (°C)**	**Mean Temperature of Warmest Quarter**	**1.9**	**3.6**
bio16 (mm)	Precipitation of Wettest Quarter	1.2	6.2
bio1 (°C)	Annual Mean Temperature	0.7	**3**
bio8 (°C)	Mean Temperature of Wettest Quarter	0.7	1.8
bio6 (°C)	Min Temperature of Coldest Month	0.6	0.1
bio5 (°C)	Max Temperature of Warmest Month	0.3	0.7
bio14 (mm)	Precipitation of Driest Month	0.2	1.1
bio17 (mm)	Precipitation of Driest Quarter	0.1	0

bio13 (mm)	Precipitation of Wettest Month	0.1	0
h_{Mean} (m)	$Altitude_{Mean}$	0	0.1

Note: Ecological predictors left after removal of predictors with high pair correlation are marked in bold.

Table S2. Correlation analysis of the environmental variables.

	bio1	bio2	bio3	bio4	bio5	bio6	bio7	bio8	bio9	bio10	bio11	bio12	bio13	bio14	bio15	bio16	bio17	bio18	bio19	h_{mean}
bio1																				
bio2	0,54																			
bio3	0,77	0,83																		
bio4	-0,76	-0,44	-0,84																	
bio5	0,81	0,42	0,41	-0,26																
bio6	0,95	0,38	0,74	-0,86	0,68															
bio7	-0,69	-0,20	-0,70	0,96	-0,19	-0,85														
bio8	0,83	0,42	0,46	-0,35	0,91	0,71	-0,28													
bio9	0,90	0,40	0,68	-0,77	0,68	0,90	-0,72	0,67												
bio10	0,86	0,41	0,45	-0,33	0,99	0,73	-0,27	0,93	0,72											
bio11	0,96	0,49	0,82	-0,89	0,66	0,99	-0,85	0,70	0,90	0,72										
bio12	0,24	0,25	0,44	-0,45	-0,07	0,27	-0,41	-0,01	0,20	0,00	0,31									
bio13	0,11	0,39	0,45	-0,37	-0,22	0,08	-0,27	-0,10	0,02	-0,14	0,17	0,88								
bio14	0,34	-0,11	0,15	-0,31	0,30	0,46	-0,39	0,22	0,39	0,30	0,39	0,48	0,04							
bio15	-0,14	0,44	0,23	0,00	-0,29	-0,27	0,15	-0,18	-0,24	-0,25	-0,15	-0,07	0,37	-0,84						
bio16	0,11	0,36	0,44	-0,38	-0,24	0,08	-0,28	-0,13	0,03	-0,16	0,17	0,90	1,00	0,07	0,34					
bio17	0,36	-0,09	0,16	-0,32	0,32	0,47	-0,39	0,24	0,40	0,32	0,40	0,50	0,07	1,00	-0,83	0,09				
bio18	0,10	0,36	0,44	-0,38	-0,25	0,08	-0,29	-0,13	0,02	-0,17	0,17	0,89	1,00	0,07	0,34	1,00	0,09			
bio19	0,36	-0,07	0,12	-0,27	0,36	0,43	-0,31	0,26	0,47	0,35	0,37	0,43	0,00	0,93	-0,80	0,03	0,94	0,03		

h_{mean}	-0,17	0,30	0,36	-0,31	-0,60	-0,15	-0,23	-0,48	-0,12	-0,54	-0,06	0,52	0,70	-0,24	0,58	0,72	-0,24	0,71	-0,26	
$h_{max-min}$	0,09	-0,04	0,24	-0,34	-0,22	0,18	-0,40	-0,15	0,12	-0,16	0,18	0,38	0,33	0,03	0,10	0,36	0,03	0,34	-0,01	0,48

Note: If two variables had r >±0.8, only one of them was selected in the same model;
